# Reticular Coordination Induced Interfacial Interstitial Carbon Atoms on Ni Nanocatalysts for Highly Selective Hydrogenation of Bio-Based Furfural under Facile Conditions

**DOI:** 10.3390/nano13020285

**Published:** 2023-01-10

**Authors:** Dandan Liu, Qiuju Fu, Chao Feng, Taisan Xiang, Han Ye, Yuting Shi, Liangjun Li, Pengcheng Dai, Xin Gu, Xuebo Zhao

**Affiliations:** State Key Laboratory of Heavy Oil Processing, College of New Energy, College of Chemistry and Chemical Engineering, China University of Petroleum (East China), Qingdao 266580, China

**Keywords:** Ni catalyst, furfural hydrogenation, tetrahydrofurfuryl alcohol, metal-organic frameworks, interstitial atom

## Abstract

A rational design of transition metal catalysts to achieve selective hydrogenation of furfural (FFR) to tetrahydrofurfuryl alcohol (THFA) under facile conditions is a promising option. In this work, a series of Ni catalysts were synthesized by controlled thermal treatment of Ni-based metal-organic frameworks (MOFs), with the purpose of modulating the interface of nickel nanoparticles by the reticular coordination in MOF precursors. The catalytic performance indicates that Ni/C catalyst obtained at 400 °C exhibits efficient conversion of FFR (>99%) and high selectivity to THFA (96.1%), under facile conditions (80 °C, 3 MPa H_2_, 4.0 h). The decomposition of MOF at low temperatures results in highly dispersed Ni^0^ particles and interfacial charge transfer from metal to interstitial carbon atoms induced by coordination in MOF. The electron-deficient Ni species on the Ni surface results in an electropositive surface of Ni nanoparticles in Ni/C-400, which ameliorates furfural adsorption and enhances the hydrogen heterolysis process, finally achieving facile hydrogenation of FFR to THFA.

## 1. Introduction

To cope with the surging demand for chemicals and carbon abatement, extensive efforts have been devoted to developing efficient catalytic systems for biomass transformation into valuable biofuels and biochemicals [1,2]. Furfural (FFR) is an imperative biomass platform on a commercial scale, derived from the hydrolysis and defunctionalization of hemicelluloses, and is also a versatile starting material that can bridge the conversion of biomass into high-accessibility value chemicals with chain lengths from C4 to C5 [3,4,5,6]. Among these useful chemical products, tetrahydrofurfuryl alcohol (THFA) is considered a capable and environmentally acceptable solvent due to its high boiling point, miscibility with water, low toxicity and biodegradability. Therefore, THFA has wide applications as a green solvent or intermediate in agriculture, printing inks and industrial or electronics cleaners [7,8]. In addition, THFA can be further transformed into economically valuable furan chemicals or 1,2- and 1,5-pentanediol by selective hydrogenation [9,10,11]. Hence, developing catalysts to realize selective conversion of FFR to THFA with high performance is greatly desired.

Conventionally, THFA is synthesized by a two-step procedure using furfuryl alcohol (FA) as in intermediate over a Cu-Cr catalyst and then Ni or noble metal catalysts separately [7]. This process achieves high THFA yields, whereas the drawbacks of this strategy, such as energy intensity, prolonged reaction times, and the toxicity of the Cu-Cr catalyst, et al., cannot be neglected. The attempt to realize direct hydrogenation of FFR to THFA in one-pot by group VIII metal catalysts (Ni, Ru, Rh, Pd and Ir) is under exploration [12,13,14]. Some research groups have found that noble metal catalysts possess high catalytic activity and could realize direct formation of THFA under mild conditions through FFR hydrogenation [12,15]; however, the high cost and limited availability restrict their large-scale application. Therefore, selectively hydrogenating FFR into THFA with comparable performance under facile conditions by a non-noble catalytic system has garnered significant attention. Among alternative non-noble metals, a Ni-based catalyst system displays acceptable activity in the hydrogenation of FFR to THFA in one-pot and tends to be a cost-effective alternative for this process [16,17]. However, to reach high yields of THFA, the optimized reaction parameters of Ni-based catalysts are strict, such as opting for a high reaction temperature or a high H_2_ pressure [16,17,18,19].

As reported, to enhance the hydrogenation selectivity of THFA, the adsorption configuration of furfural on the metal surface is crucial [20]. The flat adsorption mode of furfural is formed by both the furan ring and –CHO group interacting with the metal surface, which is beneficial for the formation of THFA. Whereas a tilted adsorption configuration on metals through the carbonyl group results in a high selectivity for FA [21,22]. In addition, the aromatic properties of the electron-rich furan ring stemming from the π bond constituted by C=C and oxygen are unfavorable for the hydrogenation of the furan ring in furfural, resulting in the harsh reaction conditions during FFR hydrogenation to THFA. To realize oriented conversion of FFR into THFA or FA, a series of tactical methods were utilized to modulate the properties of Ni, such as tuning exposed facets, controlling size, or forming intermetallics with other metals [16,18,22,23,24]. Yang et al. [20] found that Ni-Sn intermetallic compounds could realize selective hydrogenation of FFR to FA through the vertical adsorption configuration of furfural molecules, because the electron transfer from Sn to Ni could boost the activation and adsorption of C=O bonds on Ni sites while inhibiting the adsorption of C=C. Wei’s group [16] reported that the different exposed Ni facets could facilitate the selective adsorption of the furan ring or the C=O group, and finally, the selectivity of furfural hydrogenation can be switched by tuning the exposed facet of the Ni catalyst. Apart from the above strategies, interfacial modulation, especially the charge transfer at the metal-support interface, is also an important factor in reactivity [25]. The charge transfer between metal nanoparticles (NPs) and interfacial support atoms can tune the electronic and chemical properties of metal particles. The change in electronic structure can impact the adsorption and activation behavior of active sites, and eventually affect the reactivity and selectivity of catalysts. Yang et al. [26] revealed that the occurrence of electron transfer from Ni to N in Ni–N–C catalysts could boost the adsorption of nitrobenzene and decrease the H_2_ adsorption energy on Ni sites while enhancing the catalytic activity. Zhang’s group [27] evidenced that the introduced interstitial carbon atoms within the Ni lattice in the Ni_3_ZnC_0.7_/oCNT catalyst could form electron-deficient Ni with a positive charge, thereby improving the selectivity in acetylene hydrogenation. Hence, the modulation of the interface between Ni NPs and support to enhance the flat adsorption configuration of furfural is a strategy to activate both the furan ring and formyl group of FFR, which eventually improves the selectivity to THFA and softens the reaction parameters. To realize the interfacial modulation of catalysts, the structure and interaction of precursors are unneglectable; therefore, considerable attention is focused on the selection and construction of precursors.

Metal organic frameworks (MOFs) are an original precursor and template to fabricate metal oxides, metals or porous carbon via the direct pyrolysis process [28,29,30]. The uniform metal clusters and the interaction with ligands can result in homogenous metal nanoparticles combined with carbon support by special interaction, forming promising active species with suitable catalytic structures and high catalytic performance. Zhao’s group [31] demonstrated that Co NPs with Co–N_x_ active sites derived from ZIF-67 could serve as highly active and stable catalysts for selective hydrogenation of various biomass-derived organic compounds. Wang’s group [32] synthesized a Ni/C catalyst by direct thermal decomposition of Ni-BTC, which could realize the transformation of FFR to THFA, and the facilitation effect of uniform Ni NPs with small sizes was discussed. Although the pyrolysis of MOF has been used in many catalytic systems, the high temperature (≥500 °C) adopted during the pyrolysis leads to severe collapse of MOF skeletons. The intrinsic advantage of metal clusters and the coordination effect between metal and ligand in MOF are concealed completely during the pyrolysis at high temperatures. Consequently, the status of metal nanoparticles and metal-carbon interactions derived from MOF at low treatment temperatures is still unknown.

Herein, enlightened by the superiority of self-sacrificing templates of MOF, a Ni/C catalyst derived from MOF-74-Ni (CPO-27-Ni) at a low treatment temperature was obtained to explore the impact of coordination on the metal-support interface. Initially, a green hydrothermal synthetic method of MOF-74-Ni with unique and fitted size was proposed. Then, the catalytic performance of derived Ni-based catalysts on the direct transformation of FFR to THFA was evaluated. In addition, the structural advantages and Ni-C interface inherited from MOF, as well as their effect on FFR hydrogenation, were investigated by XRD, TEM, XPS, N_2_ sorption, H_2_-TPD and pulsed H_2_ chemisorption measurements. Density functional theory (DFT) calculations confirmed that the interstitial carbon atoms could change the electronic structure of Ni and that FFR was more prone to adopting a flat adsorption orientation on an electropositive Ni surface. Ultimately, the reaction parameters were optimized, and the reusability of the catalyst was checked.

## 2. Experimental Section

### 2.1. Materials

2,5-Dihydroxyterephthalic acid (H_2_dhtp) and diethyl 2,5-dihydroxyterephthalate (Et_2_dhtp) were purchased from Aladdin Reagent Co (Shanghai, China). Nickel(II) acetate tetrahydrate (Ni(COOCH_3_)_2_·4H_2_O, Ni(OAc)_2_·4H_2_O, 98%), ethanol and furfuryl alcohol were provided by Sinopharm Chemical Reagent Co (Shanghai, China). Furfural was obtained from TCI Shanghai chemicals (Shanghai, China). All the chemicals were used as received, without further purification.

### 2.2. Synthesis of MOF and Derived Catalysts

MOF-74-Ni was synthesized by a modified green hydrothermal method without using organic solvents, which is more convenient to synthesize. Typically, calculated amounts of Ni(OAc)_2_·4H_2_O (1.99 g), H_2_dhtp (0.66 g) and Et_2_dhtp (0.15 g) were mixed in 50 mL deionized water and stirred for 15 min. Then the mixture was transferred into a 100-mL Teflon-lined stainless-steel autoclave and kept at 160 °C for 5 h. The obtained mustard yellow product was centrifuged, washed three times with hot deionized water and ethanol to remove the unreacted reactants, and finally dried at 80 °C overnight, obtaining the MOF-74-Ni precursor.

The as-synthesized MOF-74-Ni precursor was transferred into a tubular furnace. Before heating, the air in the quartz tube was drained out by aerating Ar (purity > 99.99%) gas. Then, the precursor was heated to target temperature at a rate of 5 °C/min and kept for 3 h under constant Ar flow (50 mL/min). Only when the temperature of the furnace and sample were cooled to room temperature at a rate of 3 °C/min under the protection of an inert atmosphere, the calcined Ni/C catalysts could be taken out for further characterization and catalytic evaluation. The obtained samples at different temperatures were labeled Ni/C-T.

The recyclability of used catalysts was evaluated as follows. The catalyst from the last reaction was first separated from the reaction fluid by centrifugation, then washed three times with ethanol to remove the reactant and products, and finally dried in the oven for 5 h at 60 °C for the next reaction cycle.

The precursor of the Ni/C-400-G catalyst was synthesized by a physical grinding method. Briefly, 1.99 g Ni(Ac)_2_·4H_2_O and 0.78 g H_2_dhtp were intensively mixed in an agate mortar without forming coordination between Ni^2+^ and the ligand. Then the precursor was placed in a tubular furnace and treated under the same pyrolysis conditions as Ni/C-400, and the obtained sample was noted as Ni/C-400-G.

### 2.3. Characterizations

X-ray diffraction (XRD) patterns were collected on a Panalytical X’Pert PRO MPD X-ray diffractometer (Panalytical, Almelo, The Netherlands) using Cu Kα radiation. Transmission electron microscopy (TEM) was carried out on a JEM-2100UHR instrument (JEOL Ltd., Akishima, Tokyo, Japan) operating at 200 kV. The textural properties of the samples were collected by nitrogen adsorption–desorption isotherms at −196 °C on a Quantachrome Autosorb-iQ volumetric adsorption apparatus (Quantachrome Instruments, Boynton Beach, FL, USA). Before measurements, the MOF-74 sample was degassed under vacuum at 150 °C for 8 h, while the Ni/C catalysts were pretreated under a dynamic vacuum at 250 °C overnight. The pore size distribution of samples was calculated from the desorption branch of the isotherms using non-local density functional theory (NLDFT). X-ray photoelectron spectroscopy (XPS) was recorded on a Thermo Scientific ESCALAB 250Xi apparatus (Thermo Fisher Scientific, Waltham, MA, USA), and the peak fitting was achieved using Thermo Scientific Avantage software. H_2_-temperature-programmed desorption (H_2_-TPD) and pulsed H_2_ chemisorption were conducted with a Builder PCA-1200 chemical adsorption instrument (Builder, Beijing, China). For the H_2_-TPD, a 0.100-g sample was first treated in a 10% H_2_/Ar atmosphere at 300 °C for 100 min prior to the adsorption step. Then the sample was cooled to 80 °C under H_2_/Ar flow and kept at 80 °C for 40 min until the surface was saturated by H_2_. Subsequently, the samples were purged with flowing Ar (30 mL/min) to remove the weakly bound physisorbed H_2_ until the baseline was stable. The sample was heated to 400 °C at a heating rate of 10 °C/min in Ar flow and maintained at 400 °C until the signal returned to baseline. The signal of H_2_ desorption was recorded by a thermal conductivity detector (TCD). For the pulsed H_2_ chemisorption, the sample (0.100 g) was reduced at 300 °C for 100 min (10% H_2_/Ar), then the gas was switched to pure Ar, kept at 300 °C for another 30 min, and then cooled to 80 °C. Then a 10% H_2_/Ar pulse was injected into the reduced catalyst bed using Ar as a carrier gas at 80 °C. Pulses were introduced by a calibrated sample loop (V_loop_ = 0.2 mL). The adsorbed amount of H_2_ and resulting specific Ni surface area were calculated from the number and area of the adsorbed pulses and Equation (1) [33],

(1) $$S_{\mathrm{Ni}}=\frac{n_{H}\times N_{A}\times z}{m_{\mathrm{cat}}\times \sigma_{\mathrm{Ni}}}\;$$

in which S_Ni_ was the specific Ni surface area, n_H_ was the mole of adsorbed H_2_ obtained from pulsed H_2_ chemisorption. N_A_ was the Avogadro’s constant, and z was a stoichiometry factor, considering the dissociative chemisorption of H_2_ on Ni, the value of z was 2. m_cat_ was the mass of the catalyst in g, and σ_Ni_ represents the number of Ni atoms per unit area (1.54 × 10^19^ m^−2^). The dispersion of Ni was obtained according to Equation (2),

(2) $$D_{\mathrm{Ni}}=\frac{n_{H}\times z\times M_{\mathrm{Ni}}}{m_{\mathrm{cat}}\times w}$$

in which M_Ni_ was the relative atomic mass of Ni and w represented the weight fraction of Ni in catalysts.

### 2.4. Catalytic Assessment

To evaluate the catalytic performance of the prepared Ni/C samples, the hydrogenation of furfural was operated in a 100 mL stainless steel batch reactor (Senlong SML-100, Beijing Century Senlong, Beijing, China, Figure S1). For a typical reaction, the reactor was charged with a liquid mixture consisting of 0.6 g of furfural dissolved in 50 mL of ethanol and 0.10 g of catalyst. Prior to the reaction, the sealed reactor was purged three times with H_2_ to evacuate the air inside and pressurize H_2_ to a certain pressure. The reaction was performed at a different temperature while being stirred at 800 revolutions per minute (rpm) to eliminate the limitation of external diffusion. Around 0.25 mL of liquid samples were withdrawn at stipulated times during the reaction and were analyzed after centrifugation by Fuli gas chromatography equipped with a flame ionization detector (FID) and a capillary column. The products were identified by retention time in comparison with available standards and GC-MS. The quantitative analysis of the product was determined by the area normalization method based on the response factors of the standard chemicals.

The conversions of FFR (C_FFR_) and product selectivity (S_product_) were calculated as follows:

(3) $$C_{\mathrm{FFR}}=\frac{n_{\mathrm{FFR},0}-n_{\mathrm{FFR},t}}{n_{\mathrm{FFR},0}}\times 100\%$$

(4) $$S_{\mathrm{product}}=\frac{n_{\mathrm{product},t}}{n_{\mathrm{FFR},0}-n_{\mathrm{FFR},t}}\times 100\%$$

in which n_FFR,t_ represents the mole number of FFR in the reaction, n_FFR,0_ is the initial mole number of FFR before reaction, n_product,t_ is the mole number of each product (including FA, THFA and others) in the reaction.

### 2.5. Theoretical Calculation

The DFT calculations were performed using the Vienna ab-initio simulation package (VASP). The change-correlation function was determined by generalized gradient approximation combined with the Perdew–Burke–Ernzerhof (PBE) method. The Bader charge analysis and geometric relaxation of Ni and NiC_x_ were implemented using the VASP. The kinetic energy cutoff was set to 400 eV, and the Monkhorst–Pack grid was sampled as 4 × 4 × 4 k-points. The convergence criteria for atomic position and lattice parameters of Ni and NiC_x_ were set to 10^−4^ eV and 0.03 eV Å^−1^ in the total energy and force, respectively. A vacuum of 15 Å was used to simulate the surface under periodic boundary conditions. The surface of Ni(111) was used to compute the adsorption energies (E_ads_) of reactants, which were calculated according to the following equation.

E_ads_ = E_total_ − (E_surface_ + E_adsorbate_) (5)

where E_total_ is the system energy after adsorption; E_surface_ is the clean surface energy before adsorption and E_adsorbate_ is the energy of a free adsorbate in the gas phase.

## 3. Results and Discussion

### 3.1. Characterization of Catalysts

**Figure 1:**
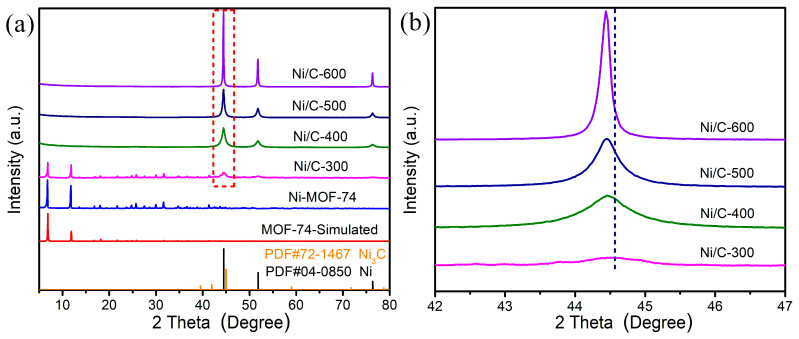
(**a**) Simulated pattern and XRD patterns of MOF-74-Ni and derived Ni/C materials treated at different temperatures, (**b**) enlarged diffraction peak attributed to the Ni (111) crystal plane in (**a**).

Initially, the nature of synthesized MOF and annealed samples is characterized by XRD to obtain structural and phase information, and the patterns are shown in Figure 1a. The XRD pattern of as-synthesized MOF-74 in the aqueous phase coincides with the simulated one, indicating successful synthesis of the MOF precursor. After thermal treatment at different temperatures, the diffraction peaks of MOF-74 gradually disappear, inferring the decomposition of the MOF skeleton. Meanwhile, three new peaks at 2θ = 44.4°, 51.7° and 76.3° are formed, which are assigned to the (111), (200) and (220) crystal planes of the pure face-centered cubic (FCC) Ni structure. The XRD pattern of Ni/C-300 demonstrates that the structure degradation of MOF-74 is initiated at around 300 °C, and the obtained sample is a mixture of MOF and metallic Ni crystallite. The broad and weak diffraction peaks of Ni indicate the metallic crystallites originated from Ni clusters in MOF with small size and poor crystallinity at 300 °C. When the temperature reaches 400 °C, the peaks attributed to MOF disappear, and only the metallic Ni^0^ phase is observed. As the temperature is further increased, more intense diffraction peaks of Ni^0^ evidence the formation of well-defined Ni crystallites with high crystallinity, and the narrower widths of the peaks indicate the gradually increased size of Ni according to the Scherrer equation. In addition, the position of the main diffraction peak attributing to the Ni (111) crystal plane shifts toward smaller angles slightly as the temperature increases (Figure 1b), indicating an expansion of the Ni crystal lattice, which may be attributed to the carbon occupation.

**Figure 2:**
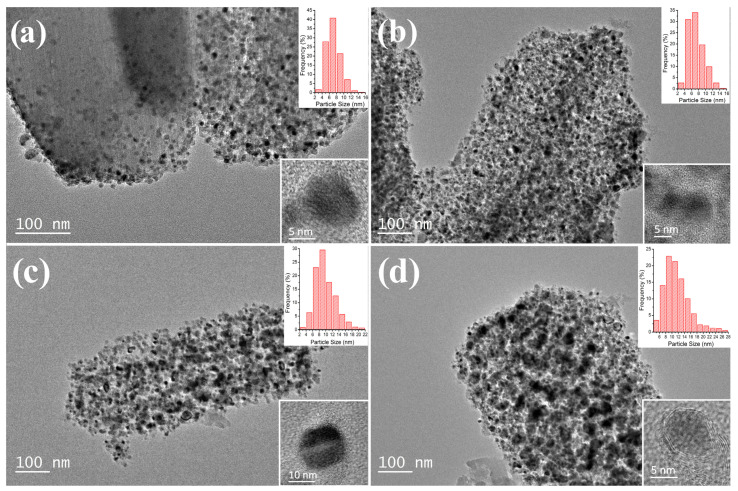
TEM images and corresponding particle size distribution of the Ni/C nanocatalysts with different treatment temperatures, (**a**) Ni/C-300, (**b**) Ni/C-400, (**c**) Ni/C-500, (**d**) Ni/C-600; inset is the magnified image of Ni^0^ particle.

Generally, the dispersion of supported nanoparticles has a close association with temperature, and the structure of catalysts and the diameter of Ni nanoparticles are investigated by TEM. From Figure 2, the obtained Ni/C samples at different temperatures still partially maintain the rod-like morphology of their MOF-74 precursors (Figure S2), and dispersed Ni NPs are observed in the matrix of carbon or undecomposed MOF frameworks. The particle size distribution is confirmed by measuring 400 particles for statistics throughout the observation of Ni/C samples. Due to the confinement in MOF frameworks, small and uniform Ni NPs are observed in Ni/C-300, but relatively sparse Ni NPs indicate only partial Ni species are reduced in the thermal treatment process. The average Ni particle size of Ni/C-400 is similar to Ni/C-300 with the aid of coordination, whereas the quantity of particles is obviously increased. In TEM images of Ni/C-500 and Ni/C-600, an increased average diameter of Ni NPs is confirmed by observation, and those enlarged particles are mainly ascribed to the agglomeration or sintering of Ni particles at higher temperatures, which is consistent with the SEM (Figure S2) results. Moreover, the formed carbon layers are not able to wrap the Ni particles entirely at 500 °C, and the well-defined carbon layers almost cover the metal surface after the temperature reaches 600 °C, which may block the surface active sites on Ni NPs and affect the adsorption of reactants. In the HRTEM images of Ni/C samples (Figure S3), only a typical lattice spacing of 0.204 nm is detected, which is assigned to the (111) plane of the FCC Ni.

Furthermore, the evolution of Ni NPs with temperature is also recorded by SAED patterns (Figure S4). For the sample treated at 300 °C, the electron diffraction pattern attributing to FCC Ni can be indexed, and the diffraction dots of MOF can also be observed in the pattern, indicating the coexistence of Ni NPs and MOF. When the annealing temperature reaches 400 °C, except for the rings attributing to Ni, a weak, broad, but recognizable diffraction ring indexed as Ni_3_C carbide (10·2) crystal plane (card JCPDS 01-072-1467) is presented, and this broad halo indicates the Ni_3_C carbide phase exists in small size [34,35]. Due to the transitional and metastable nature of Ni_3_C, the measured average interplanar spacing (d_obs_ = 1.49 Å) is inferior to the crystalline one (d = 1.56 Å). After the sample is annealed at 500 °C, the diffraction ring from Ni_3_C becomes unrecognizable due to the decomposition of Ni_3_C species [36], and the narrower diffraction rings of Ni imply the enhancement of crystallinity. As for the sample obtained at 600 °C, the obvious dots in the diffraction rings indicate the formation of Ni crystallites with increased size and higher crystallinity, in accordance with the TEM results.

**Figure 3:**
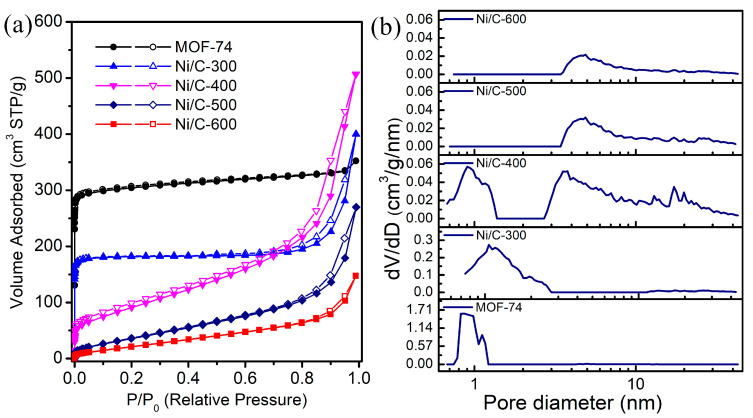
(**a**) N_2_ adsorption−desorption isotherms and (**b**) pore size distribution of MOF-74 and derived Ni/C at different temperatures.

In the catalytic process, the porous structure of samples also has a large effect on the catalytic performance. Figure 3 shows the N_2_ sorption isotherms and pore diameter distributions of MOF-74 and derived Ni/C samples at different temperatures. In addition, the detailed pore parameters of these samples are summarized in Table S1. MOF-74 exhibits typical type I isotherms, and the sharp increase near P/P_0_ = 0 in the adsorbed N_2_ isotherm is due to the existence of micropores in frameworks. The corresponding BET surface area and pore volume of MOF-74 are estimated to be 1249 m^2^·g^−1^ and 0.55 cm^3^·g^−1^, respectively, which indicates that the modified hydrothermal synthetic process can form uniform MOF-74 nanorods without sacrificing the porous structure. The pore size distribution calculated by non-local density functional theory (NLDFT) shows that the micropores are mainly concentrated between 0.75 and 1.22 nm. Ni/C-300 displays type I isotherms with a hysteresis loop at 0.75 < P/P_0_ < 0.99. This type of isotherm and pore size distribution indicate the microporous structure in MOF-74 is partially transformed into mesopores after thermal treatment at 300 °C, accompanied by the sacrifice of microporous surface area in Ni/C-300 (749 m^2^·g^−1^ and 0.62 cm^3^·g^−1^). Compared with the MOF-74 precursor, a drastic change occurs after the treatment temperature reaches 400 °C. Ni/C-400 displays a surface area of 336 m^2^·g^−1^ and the highest pore volume of 0.78 cm^3^·g^−1^. The BET surface area becomes much smaller due to structure collapse, and a more obvious characteristic hysteresis for the type IV isotherm is observed, demonstrating the formation of mesoporous structure. In the pore size distribution of Ni/C-400, except for the micropores, a new peak appears around 2.7–10.0 nm, corresponding to the formation of mesoporous, which demonstrates that Ni/C-400 possesses hierarchical structure. For Ni/C-500, the distribution of microporous materials disappears, and the diameter of mesopores is concentrated to 3.4–10 nm, suggesting a total disruption of MOF-74 structure. No micropores are confirmed in the sample of Ni/C-600 as well; in addition, the surface area and pore volume drastically decrease to only 101 m^2^·g^−1^ and 0.23 cm^3^·g^−1^, respectively.

**Figure 4:**
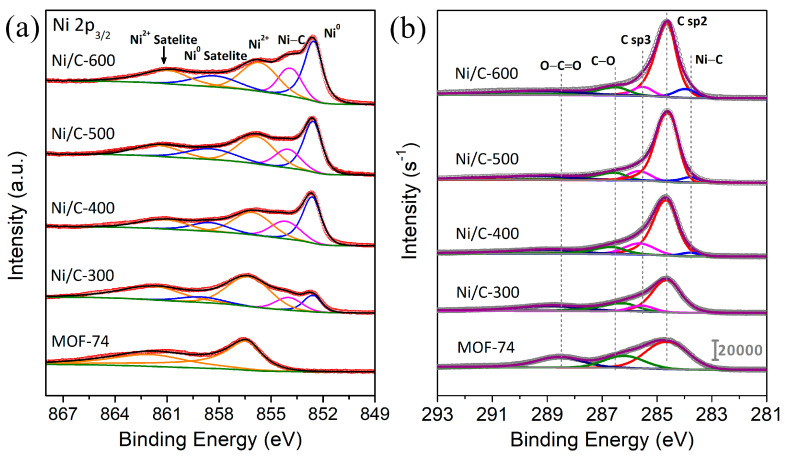
(**a**) Ni 2p3/2 XPS spectra and (**b**) C 1s XPS spectra of the MOF-74 and Ni/C derivatives prepared at various temperatures.

To obtain insight into the surface compositions and electronic states of species in derived Ni/C catalysts, X-ray photoelectron spectroscopy (XPS) is carried out. Figure S5 is the XPS survey spectra of samples, which indicates the coexistence of Ni, C and O elements before and after thermal treatment in all samples. Figure 4 displays the high-resolution XPS spectra of the Ni 2p3/2 and C 1s regions. In the high-resolution Ni 2p3/2 spectra of MOF-74 (Figure 4a), the distinct peak splits at 856.5 and 862.2 eV, corresponding to the Ni^2+^ ionic state and Ni^2+^ satellite peaks in MOF, indicating that Ni species in MOF-74 exist as Ni^2+^ in the frameworks. The XPS spectra of Ni species in the samples after treatment can be separated into five peaks with binding energies of about 852.5, 853.9, 855.9, 858.7 and 862.1 eV. The peaks at 852.5 and 858.7 eV are attributed to the metallic Ni and satellite peaks [33]. The other two main peaks with binding energies of 855.9 and 862.1 eV correspond to the Ni^2+^ and relevant satellite peaks [37]. After thermal treatment at 300 °C, the peaks derived from metallic Ni species appear, and the proportion of this peak increases with the rising treatment temperatures. The Ni^0^ content in the Ni/C-300 sample is only 17.6%, while in Ni/C-600 catalyst, the content of Ni^0^ increased to 41.1% (Table S2). However, the Ni 2p3/2 binding energy of metallic Ni in Ni/C-400 (852.6 eV) is 0.3 eV higher than the Ni metal peaks of Ni/C-300 (852.3 eV). Combined with the shift of the diffraction peak in XRD, the electronic property of Ni^0^ is modified by different treatment temperatures. Moreover, other Ni species located between Ni^0^ and Ni^2+^ are formed during the treatment process. The appearance of this new species is mainly attributed to the Ni species located at the interface between Ni NP and carbon support, and the electronic interactions between Ni and carbon support result in charge redistribution at the interfaces. The formation of this Ni species is due to the diffusion and dislocation at the interface between Ni and the carbonaceous support derived from MOF-74 and the interstitial C atoms in the metallic Ni surface, resulting in the presentation of metastable and amorphous Ni-C_x_ species [36,38]. At 300 °C, the Ni species mainly exist as Ni^2+^ in the frameworks, only 28.2% of the coordinated Ni^2+^ is reduced, and the atom ratio of interfacial Ni atoms can reach 10.6%, which has an association with small particle size and a high surface ratio. When the temperature reaches 400 °C, accompanied by the framework decomposition of MOF-74, 57.3% Ni^2+^ in the frameworks is reduced in this process, and 29.1% of the Ni species are reduced and present in the form of amorphous Ni_3_C due to the high dispersion of Ni NPs and adequate temperature. While, as the treatment temperature increases, the aggregation and growth of Ni NPs enhance the size and crystallinity of particles when the treatment temperature increases. At the same time, the metastable state Ni_3_C species between Ni NP and carbonaceous support decompose into metallic Ni and graphene [38], and the formed carbon layers gradually cover the surface of Ni NPs. The Ni-C peaks presented at high decomposition temperatures are induced by the decomposition of amorphous metastable Ni_3_C to Ni and carbon atoms, whereas the high temperature facilitates the mixing of partial carbon and Ni atoms at the boundaries of crystalline. This process eventually forms an intermediate, solid solution with a small amount of carbon solved into nickel atoms rather than the ordinary carbide phase [39].

To verify the assistance of carbon support, the C 1s XPS spectra are investigated. The evolution of the C 1s spectra with different treatment temperatures and associated component peaks fit for MOF-74 and Ni/C samples are shown in Figure 4b. As observed, the line shape of the MOF-74 spectrum can be separated into three peaks, which correspond to three kinds of carbon in the ligand. The peak at 284.6 eV is assigned to sp2 C−C bonds, while the peak at 286.2 eV can be assigned to C−O bonds, and the third peak at 288.5 eV is corresponding to the O−C=O in the ligand [37]. After heat treatment, careful peak-fitting analysis is carried out for all the C 1s spectra. When the heating temperature is elevated to 300 °C, the peak ratio attributed to C−O and O−C=O is decreased, indicating the partial destruction of the ligands hydroxyl and carboxyl during treatment. An additional peak at 285.6 eV appears in the fitting, which is associated with the sp3 hybridized carbon (sp3 C−C) originating from the decomposition of ligand [38]. At about 400 °C, the carboxyl carbon content is negligible, and the broken coordination results in an increased content of sp3 carbon (Table S3). This conversion is also accompanied by the formation of the C species at 283.8 eV, which corresponds to the interstitial carbon in the amorphous Ni_3_C species [39]. Moreover, the location of C−O shifted to 286.6 eV, suggesting the C−O sort changes from ligand phenolic hydroxyl to oxygenic groups on carbon. When continuing to increase the treatment temperature, the component assignment to sp3 hybrids C decreases in proportion, which is attributable to the graphitization of the samples at high temperatures. In addition, more carbon atoms insert into the grain boundaries of Ni, leading to an increase in the peak ratio of Ni−C. Specifically, the decomposition and carbonization of ligands at low temperatures (≤400 °C) can form an amorphous carbon phase. The structurally disordered amorphous carbon that surrounded or inserted the small grain-size Ni particles could partly bond to the interfacial Ni atoms, causing charge transfer effects from the surface Ni atoms to the carbon atoms of amorphous carbon [37]. Whereas, at temperatures higher than 400 °C, the aggregation of Ni NPs, the decomposition of the NiC_x_ component, as well as the formation of graphene layer, are not beneficial factors for the adsorption and activation of the furan ring in reactants.

### 3.2. Selective Hydrogenation of Furfural over Ni/C Catalysts

**Scheme 1:**
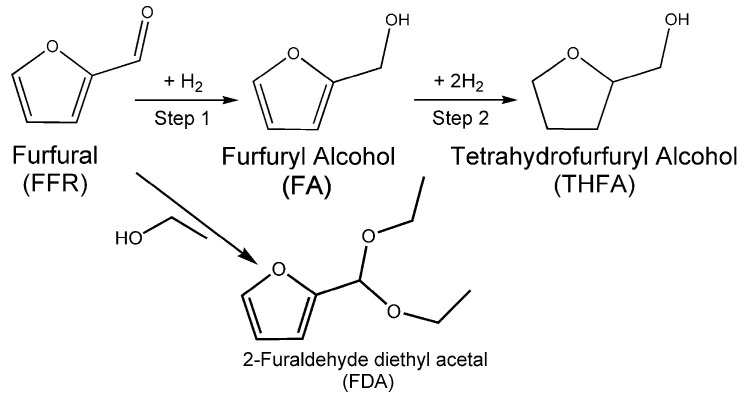
Pathways for hydrogenation of furfural (FFR) to tetrahydrofurfuryl alcohol (THFA) via furfuryl alcohol (FA) and the formation of byproduct 2-furaldehyde diethyl acetal (FDA).

To associate the relationship between structure and catalytic activity, the performance of prepared Ni/C catalysts on catalyzing furfural (FFR) hydrogenation is evaluated. The hydrogenation process of FFR is given in Scheme 1, where the generated products include furfuryl alcohol (FA), tetrahydrofurfuryl alcohol (THFA) and byproduct 2-furaldehyde diethyl acetal (FDA). Table 1 presents the catalytic performance of MOF-74 and derived Ni/C catalysts at different temperatures. Compared with the MOF-74 precursor, 99% conversion of FFR and 77.5% selectivity of THFA are obtained in the case of Ni/C-300. As for the Ni/C-400 catalyst, a considerably high conversion (99%) of FFR and enhanced selectivity (>95%) of THFA are acquired. Unfortunately, as the treatment temperature increases, the conversion of FFR remains at 99%, but the selectivity declines to 85.7% over the Ni/C-500 catalyst. Ni/C-600 exhibits an obvious reduction in conversion (82.1%) and selectivity of THFA (17.8%), but the selectivity of FA increases to 77.1%. For comparison, only 0.9% conversion is obtained in the absence of a catalyst. Moreover, when MOF-74 is used as a catalyst without any treatment, the conversion of FFR is 9.3%.

In combining the catalytic performance with the SAED and XPS results, highly active Ni NPs are created in Ni/C-400 due to the formation of highly dispersed Ni NPs with electropositive interfacial Ni species, which is beneficial for the flat adsorption of FFR with an electron-rich furan ring. Furthermore, the enhanced FA selectivity, along with the increased heat-treatment temperature, has an association with the aggregation of Ni and the decomposition of interfacial amorphous Ni_3_C species. The formed graphene layers at high treatment temperatures could cover the surface of Ni NPs and change the flat adsorption of FFR into tilted adsorption, which eventually switched the THFA/FA selectivity. Compared with the microporous structure of Ni/C-300, the micropores in Ni/C-400 can limit the aggregation of Ni NPs, and the appearance of mesopores can further lessen the mass transport limitation of molecules to active sites during reaction, improving the diffusion and frequency of contact between reactive molecules and active metal sites.

### 3.3. Characterization of the Activation Mechanism

**Figure 5:**
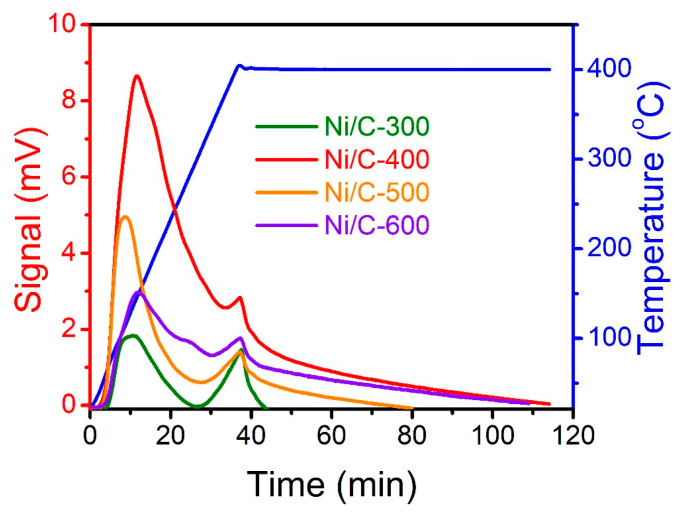
H_2_-TPD curves of the MOF-74-derived Ni/C catalysts at different temperatures.

The activation of H_2_ is a crucial process during the catalytic hydrogenation reaction, and the H_2_ dissociation process is highly sensitive to the surface Ni. Hence, for further investigating the H_2_ dissociation difference between four catalysts, the H_2_-TPD profiles of the four Ni/C catalysts are detected (Figure 5). To avoid the interference of the TCD signal caused by further decomposition of the catalyst, the temperature program is ended at 400 °C. The hydrogen desorption peaks in the range of 50–300 °C are mainly ascribed to the removal of chemisorbed hydrogen on the metal surface, and the area of the desorption peak represents the amount of desorbed hydrogen. Ni/C-400 catalyst exhibits the highest amount of chemisorbed hydrogen, which has an association with surface electron-deficient Ni species [40]. However, higher treatment temperatures lead to Ni aggregation and the formation of a carbon layer, which may cover and decrease the surface active Ni content, leading to a decline in the ability to adsorb and dissociate H_2_.

The metal surface area and particle dispersion of MOF-74-derived Ni/C catalysts are calculated by pulsed H_2_ chemisorption, and the results are listed in Table S4. The results show that both the metal surface area and particle dispersion of Ni/C-400 are notably higher than others, which is an important factor resulting in high catalytic performance. The low metal surface area and particle dispersion of Ni/C-300 are mainly attributed to the lower reduction ratio in MOF-74, although the particle size of metallic Ni is similar to Ni/C-400. While the metal surface area and particle dispersion of Ni/C decrease sharply as the treatment temperature increases from 400 °C to 600 °C. Especially for the Ni/C catalyst obtained at 600 °C, the lower Ni surface area and dispersion can decrease the ability to dissociate H_2_ and reduce FFR conversion during hydrogenation.

**Figure 6:**
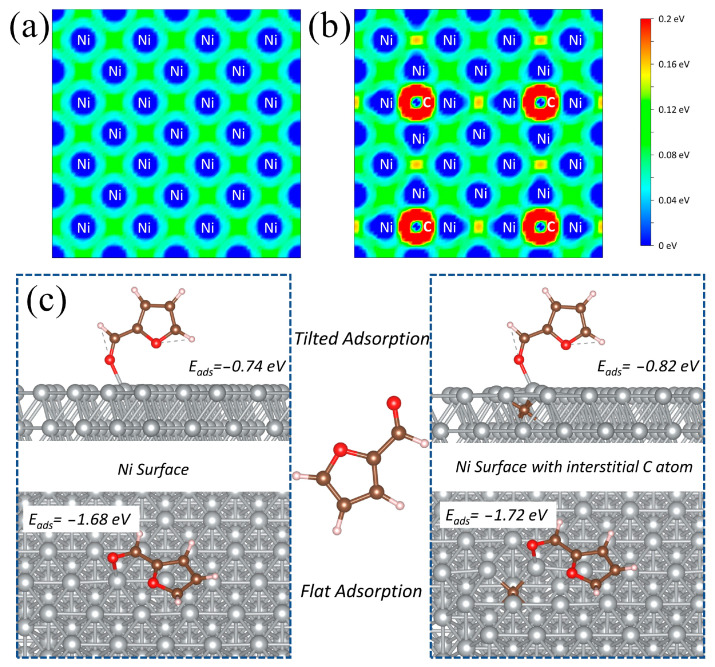
Charge density difference plots of (**a**) Ni and (**b**) Ni with interstitial carbon, (**c**) adsorption energies of different furfural adsorption configurations on the surface of Ni (111) with and without interstitial carbon.

To verify the effect of interstitial carbon atoms on the surface of Ni NP, density functional theory (DFT) calculations are performed to understand the relationship between the carbon-inserted surface and adsorption configuration. The variation of the electronic structure of Ni caused by the charge transfer between C and Ni in Ni/C-400 is demonstrated in Figure 6. The charge transfer distribution maps acquired from DFT calculations clearly display the charge density differences in Ni and Ni with interstitial carbon along the (111) directions, respectively [25]. Compared with Ni (Figure 6a), an obvious charge transfer occurs from Ni to C after C incorporation, rendering Ni with electron deficiency (Figure 6b). Bader charge analysis shows that the electric charge of Ni (top site) dramatically changes from +0.001|e| to +0.15|e| (Table S5), indicating electron transfer from the Ni atom to the incorporated carbon atom and further demonstrating the formation of electron-deficient Ni with a positive charge on the surface.

The (111) facet of Ni, as the most preferential and stable plane, [14] is chosen for adsorption calculations, and the adsorption energies of flat and tilted adsorption on pure Ni and Ni surfaces with interstitial carbon are compared. In the case of the Ni system, the FFR adsorption energies of tilted adsorption and flat adsorption are −0.74 eV and −1.68 eV, respectively (Figure 6c), indicating the preferentiality of flat adsorption. After carbon incorporation, the enhanced adsorption energy (−0.82 eV and −1.72 eV) implies improved FFR adsorption on an electron-deficient Ni surface. The higher adsorption energy (−1.72 eV) for flat adsorption indicates that furfural is more inclined to adopt flat adsorption on electropositive Ni sites of NiC_x_ with high adsorption energy and effective activation, resulting in the high selectivity of THFA. Therefore, the electropositive surface due to the presence of interstitial carbon atoms in Ni/C-400 can enhance the selectivity of THFA by improving the flat adsorption of FFR.

**Scheme 2:**
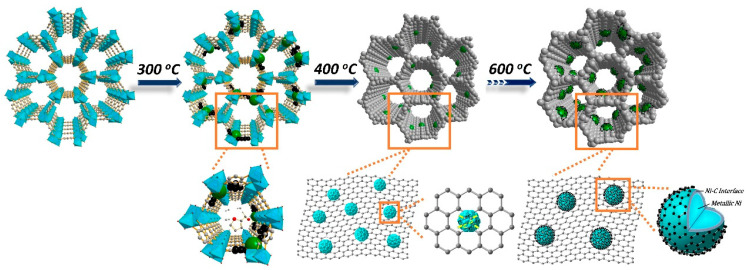
Formation of Ni nanoparticles derived from MOF-74 at different heat treatment temperatures with different surface structures.

The superior performance of catalysts has a close relationship with the structure and transformation process of MOF. When the treatment temperature is set at 300 °C, only partial Ni^2+^ is in-situ transformed into Ni NPs, reducing the quantity and density of active sites inevitably. After the temperature reaches 400 °C, the frameworks of MOF-74 decompose at this stage, accompanied by the transformation of Ni^2+^ into metallic Ni NPs in the reductive atmosphere. At the same time, because of the removal of functional groups, partial sp2 hybridized carbon atoms gradually change into sp3 hybridized carbon atoms, resulting in the formation of amorphous carbon support. Concerning the Ni−C interface, the breakage of hydroxyl and coordinated carboxyl in the ligand could cause the formation of metastable amorphous NiC_x_, due to the interstitial carbon atoms and bridge carbon atoms partly bonded to surface nickel atoms. The charge transfer in the NiC_x_ phase results in an electron-deficient electropositive surface on the Ni active phase, hence improving the activation of hydrogen and enhancing the flat adsorption ratio of the electron-rich furan ring in furfural. When the treatment temperature increases to 500 °C or even 600 °C, the formed NiC_x_ species in the interface decomposed. Moreover, the generated carbon shells partially cover the surface active sites and change the adsorption configuration to tilted adsorption, resulting in a change in the selectivity of the main product from THFA to FA (Scheme 2). To verify the importance of the reticular coordination effect in MOF, the raw materials used for the synthesis of MOF-74-Ni were first mixed homogeneously by physical grinding, then treated under the same conditions with Ni/C-400. The results demonstrate that although most ligands are volatilized during this process, the obtained Ni/C-400-G catalyst is also a Ni/C catalyst (Figure S6). However, the Ni particles in Ni/C-400-G present a larger particle size with high crystallinity (Figure S7). Furthermore, the catalytic performance of Ni/C-400-G exhibits a noticeable disparity with Ni/C-400 (Table S6). Therefore, it is believed that the high catalytic activity and selectivity of Ni/C-400 for the total hydrogenation of FFR to THFA (Table S7) are closely related to the higher reduction ratio of Ni species and the electronically defective surface of well-dispersed Ni NPs induced by the reticular coordination of MOFs.

### 3.4. Effect of Reaction Conditions and Recycling Test

**Figure 7:**
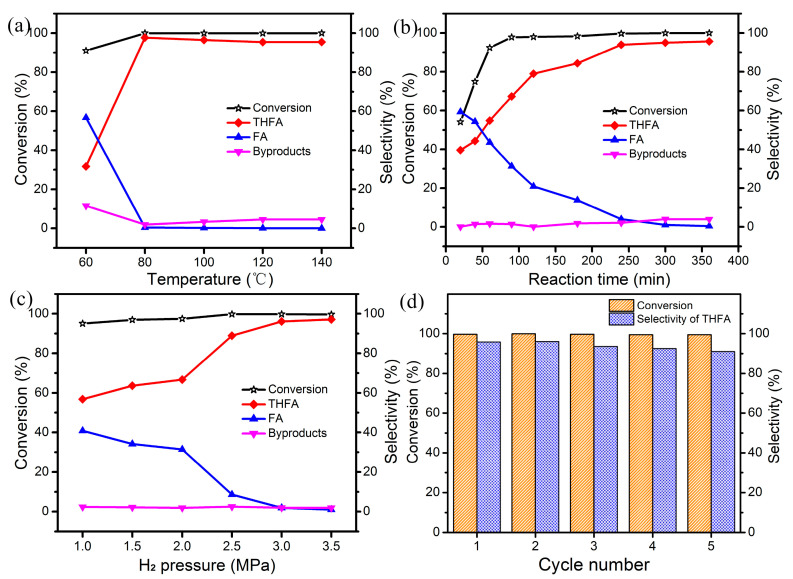
(**a**) effect of reaction temperature: 3 MPa, 5 h; (**b**) effect of reaction time: 80 °C, 3 MPa; (**c**) effect of reaction H_2_ pressure: 80 °C, 4 h; (**d**) the reusability of catalyst: 80 °C, 3 MPa, 4 h; other reaction conditions: 0.6 g FFR, 50 mL ethanol, 0.1 g catalyst: Ni/C-400.

To gain more insight into the hydrogenation process of FFR to THFA over Ni/C-400, different reaction parameters such as reaction temperature, H_2_ pressure and reaction time are optimized, which are illustrated in Figure 7. The temperature of reaction is explored within 60–140 °C (Figure 7a), where an obvious increase from 31.7% to 97.7% is observed for the selectivity of THFA from 60 to 80 °C, but the selectivity of FA presents an opposite trend. When further increasing the reaction temperature to 140 °C, the selectivity of THFA starts to decrease, and the ratio of by-products gradually increases. Therefore, 80 °C is an appropriate temperature for the hydrogenation of FFR over Ni/C-400. The variation of conversion and product distribution with time is evaluated at 80 °C and 3 MPa H_2_ pressure (Figure 7b). The conversion increases sharply from 54.1% to 97.8% before 80 min and keeps steady after 80 min, while the selectivity to THFA achieves balance after 240 min (4 h). Prolonging the reaction time to 360 min, there is no apparent variation and improvement in the selectivity of THFA, and byproducts such as FDA are formed [16]. Thus, 4 h is the desired reaction time for Ni/C-400 at 80 °C. Additionally, the effect of H_2_ pressure is studied at 80 °C and 4 h (Figure 7c). When enhancing the H_2_ pressure from 1 MPa to 2.5 MPa, the selectivity of FA and other byproducts drops from 43.2% to 11.1%. While at 3 MPa, the selectivity of THFA can reach 96.1%, and no longer enhances with the increase in H_2_ pressure. Hence, 80 °C and an initial H_2_ pressure at 3 MPa for 4 h are proper reaction conditions for the total hydrogenation of FFR to THFA in ethanol over Ni/C-400.

Catalytic recyclability is one of the critical factors for heterogeneous catalysts, and the recycling experiment of Ni/C-400 is performed at optimum reaction conditions, which is shown in Figure 7d. After being recycled five times, the conversion of FFR remains unchanged (higher than 99%), and the selectivity of THFA exhibits a slight decrease. The slight decrease in catalytic activity is probably due to the products’ deposition on the surface of Ni NPs. These results demonstrate that the Ni/C-400 possesses acceptable recyclability under suitable catalytic conditions.

## 4. Conclusions

In summary, a series of Ni/C catalysts have been synthesized by controlled decomposition of MOF at different temperatures, and the interfacial difference of Ni nanoparticles induced by reticular coordination in MOF results in an apparent discrepancy in catalytic performance in the hydrogenation of furfural (FFR) to tetrahydrofurfuryl alcohol (THFA). Among these catalysts, the Ni/C catalyst obtained at 400 °C presents higher catalytic activity, great selectivity for THFA and steady recyclability at facile reaction conditions. The prominent catalytic performance of Ni/C-400 stems from the highly dispersed metallic Ni nanoparticles and the electronically deficient surface, which is beneficial for the flat adsorption of furfural. The electropositive Ni surface evidenced by DFT calculations is induced by charge transfer from Ni nanoparticles to interfacial and interstitial carbon atoms of carbonaceous support during the decomposition of MOF, and the highly dispersed metallic Ni nanoparticles can enhance the hydrogen dissociation. In addition, the catalytic condition of Ni/C-400 is optimized, and the direct production of THFA by FFR can be realized at a mild condition (80 °C, 3 MPa of H_2_ pressure, 4 h). The special interaction between metal and support derived from MOF at low treatment temperatures, provides a new strategy for modifying the surface electronic structure of metal catalysts and accelerating the development of non-noble catalysts in various catalysis processes.

## Figures and Tables

**Table 1:** The catalytic performance of MOF-74 and derived Ni/C catalysts.

Entry	Catalyst	Temperature	H_2_ Pressure	Time	Conversion	Selectivity (%)		
		(°C)	(MPa)	(h)	(%)	FA	THFA	Others
1	None	120	3	5	0.9	0	0	100
2	MOF-74	120	3	5	9.3	46.0	44.9	9.1
3	Ni/C-300	120	3	5	>99	8.4	77.5	14.1
4	Ni/C-400	120	3	5	>99	0.1	97.7	2.2
5	Ni/C-500	120	3	5	>99	7.0	85.7	7.3
6	Ni/C-600	120	3	5	82.1	77.1	17.8	5.1

Reaction conditions: 0.6 g FFR, 0.1 g catalysts, 50 mL ethanol. Others: refer to byproducts mainly containing 2-furaldehyde diethyl acetal and others not detected.

## Data Availability

The data is available on reasonable request from the corresponding author.

## Supplementary Materials

The following supporting information can be downloaded at: https://www.mdpi.com/article/10.3390/nano13020285/s1, Figure S1: The apparatus of stainless steel batch reactor for catalyst performance evaluation of furfural hydrogenation; Figure S2: SEM images of MOF-74-Ni and derived Ni/C catalysts: (a) MOF-74-Ni, (b) Ni/C-300, (c) Ni/C-400, (d) Ni/C-500, (e) Ni/C-600; Figure S3: HRTEM images of Ni/C catalysts derived from MOF-74-Ni at different temperatures: (a) Ni/C-300, (b) Ni/C-400, (c) Ni/C-500, (d) Ni/C-600; Figure S4: Electron diffraction patterns of Ni/C catalysts derived from MOF-74-Ni at different temperatures: (a) Ni/C-300, (b) Ni/C-400, (c) Ni/C-500, (d) Ni/C-600; Figure S5: XPS survey spectra of the MOF-74 and Ni/C derivative prepared at different temperatures; Figure S6: (a) XRD patterns of mixture of Ni(Ac)∙4H_2_O and H_2_dhtp by physical grinding, and (b) comparison of XRD patterns between Ni/C-400 and Ni/C-400-G; Figure S7: (a–c) TEM images of Ni/C-400-G catalyst with different magnifications, and d) corresponding particle size distribution; Table S1: Characteristics of Ni-MOF-74 and Ni/C catalysts with different treatment temperatures; Table S2: XPS Data for the MOF-74 and Ni/C derivative prepared at different temperatures; Table S3: C1s XPS Data for the MOF-74 and Ni/C derivative prepared at different temperatures; Table S4: The physicochemical properties of Ni/C nanocatalysts obtained at different temperatures; Table S5: The Bader charge analysis of (a) Ni and (b) Ni with interstitial carbon; Table S6: The catalytic performance of Ni/C-400 and Ni/C-400-G; Table S7: Summary for hydrogenation of furfural to tetrahydrofurfuryl alcohol from literatures [13,15,16,17,18,22,24,30,32,41,42,43,44].

## References

[1] Ahmad F.B., Zhang Z., Doherty W.O.S., O’Hara I.M. (2019). The outlook of the production of advanced fuels and chemicals from integrated oil palm biomass biorefinery. Renew. Sust. Energ. Rev..

[2] Gallezot P. (2012). Conversion of biomass to selected chemical products. Chem. Soc. Rev..

[3] Nakagawa Y., Tamura M., Tomishige K. (2013). Catalytic Reduction of Biomass-Derived Furanic Compounds with Hydrogen. ACS Catal..

[4] Sun D., Sato S., Ueda W., Primo A., Garcia H., Corma A. (2016). Production of C4 and C5 alcohols from biomass-derived materials. Green Chem..

[5] Bohre A., Dutta S., Saha B., Abu-Omar M.M. (2015). Upgrading Furfurals to Drop-in Biofuels: An Overview. ACS Sustain. Chem. Eng..

[6] Jin Z., Yi X., Wang L., Xu S., Wang C., Wu Q., Wang L., Zheng A., Xiao F.-S. (2019). Metal-acid interfaces enveloped in zeolite crystals for cascade biomass hydrodeoxygenation. Appl. Catal. B.

[7] Yan K., Wu G., Lafleur T., Jarvis C. (2014). Production, properties and catalytic hydrogenation of furfural to fuel additives and value-added chemicals. Renew. Sustain. Energy Rev..

[8] Tike M.A., Mahajani V.V. (2007). Kinetics of Liquid-Phase Hydrogenation of Furfuryl Alcohol to Tetrahydrofurfuryl Alcohol over a Ru/TiO2 Catalyst. Ind. Eng. Chem. Res..

[9] Soghrati E., KokPoh C., Du Y., Gao F., Kawi S., Borgna A. (2018). C−O Hydrogenolysis of Tetrahydrofurfuryl Alcohol to 1,5-Pentanediol Over Bi-functional Nickel-Tungsten Catalysts. ChemCatChem.

[10] Koso S., Ueda N., Shinmi Y., Okumura K., Kizuka T., Tomishige K. (2009). Promoting effect of Mo on the hydrogenolysis of tetrahydrofurfuryl alcohol to 1,5-pentanediol over Rh/SiO_2_. J. Catal..

[11] Li L., Barnett K.J., McClelland D.J., Zhao D., Liu G., Huber G.W. (2019). Gas-phase dehydration of tetrahydrofurfuryl alcohol to dihydropyran over γ-Al_2_O_3_. Appl. Catal. B..

[12] Huang R., Cui Q., Yuan Q., Wu H., Guan Y., Wu P. (2018). Total Hydrogenation of Furfural over Pd/Al_2_O_3_ and Ru/ZrO_2_ Mixture under Mild Conditions: Essential Role of Tetrahydrofurfural as an Intermediate and Support Effect. ACS Sustain. Chem. Eng..

[13] Bhogeswararao S., Srinivas D. (2015). Catalytic conversion of furfural to industrial chemicals over supported Pt and Pd catalysts. J. Catal..

[14] Li S., Wang Y., Gao L., Wu Y., Yang X., Sheng P., Xiao G. (2018). Short channeled Ni-Co/SBA-15 catalysts for highly selective hydrogenation of biomass-derived furfural to tetrahydrofurfuryl alcohol. Micropor. Mesopor. Mater..

[15] Nguyen-Huy C., Kim J.S., Yoon S., Yang E., Kwak J.H., Lee M.S., An K. (2018). Supported Pd nanoparticle catalysts with high activities and selectivities in liquid-phase furfural hydrogenation. Fuel.

[16] Meng X., Yang Y., Chen L., Xu M., Zhang X., Wei M. (2019). A Control over Hydrogenation Selectivity of Furfural via Tuning Exposed Facet of Ni Catalysts. ACS Catal..

[17] Gong W., Chen C., Zhang H., Wang G., Zhao H. (2018). Highly dispersed Co and Ni nanoparticles encapsulated in N-doped carbon nanotubes as efficient catalysts for the reduction of unsaturated oxygen compounds in aqueous phase. Catal. Sci. Technol..

[18] Wu J., Gao G., Li J., Sun P., Long X., Li F. (2017). Efficient and versatile CuNi alloy nanocatalysts for the highly selective hydrogenation of furfural. Appl. Catal B.

[19] Jia P., Lan X., Li X., Wang T. (2018). Highly Active and Selective NiFe/SiO_2_ Bimetallic Catalyst with Optimized Solvent Effect for the Liquid-Phase Hydrogenation of Furfural to Furfuryl Alcohol. ACS Sustain. Chem. Eng..

[20] Chen S., Wojcieszak R., Dumeignil F., Marceau E., Royer S. (2018). How Catalysts and Experimental Conditions Determine the Selective Hydroconversion of Furfural and 5-Hydroxymethylfurfural. Chem. Rev..

[21] Yang Y., Chen L., Chen Y., Liu W., Feng H., Wang B., Zhang X., Wei M. (2019). The selective hydrogenation of furfural over intermetallic compounds with outstanding catalytic performance. Green Chem..

[22] Tang F., Wang L., Dessie Walle M., Mustapha A., Liu Y.-N. (2020). An alloy chemistry strategy to tailoring the d-band center of Ni by Cu for efficient and selective catalytic hydrogenation of furfural. J. Catal..

[23] Han J.W., Park J.S., Choi M.S., Lee H. (2017). Uncoupling the size and support effects of Ni catalysts for dry reforming of methane. Appl. Catal. B.

[24] Liu L., Lou H., Chen M. (2018). Selective hydrogenation of furfural over Pt based and Pd based bimetallic catalysts supported on modified multiwalled carbon nanotubes (MWNT). Appl. Catal. A.

[25] van Deelen T.W., Hernández Mejía C., de Jong K.P. (2019). Control of metal-support interactions in heterogeneous catalysts to enhance activity and selectivity. Nat. Catal..

[26] Yang F., Wang M., Liu W., Yang B., Wang Y., Luo J., Tang Y., Hou L., Li Y., Li Z. (2019). Atomically dispersed Ni as the active site towards selective hydrogenation of nitroarenes. Green Chem..

[27] Niu Y., Huang X., Wang Y., Xu M., Chen J., Xu S., Willinger M.-G., Zhang W., Wei M., Zhang B. (2020). Manipulating interstitial carbon atoms in the nickel octahedral site for highly efficient hydrogenation of alkyne. Nat. Commun..

[28] Chen Y.-Z., Zhang R., Jiao L., Jiang H.-L. (2018). Metal–organic framework-derived porous materials for catalysis. Coord. Chem. Rev..

[29] Dang S., Zhu Q.-L., Xu Q. (2017). Nanomaterials derived from metal–organic frameworks. Nat. Rev. Mater..

[30] Wu J., Zhang X., Chen Q., Chen L., Liu Q., Wang C., Ma L. (2020). One-Pot Hydrogenation of Furfural into Tetrahydrofurfuryl Alcohol under Ambient Conditions over PtNi Alloy Catalyst. Energy Fuels.

[31] Gong W., Lin Y., Chen C., Al-Mamun M., Lu H.-S., Wang G., Zhang H., Zhao H. (2019). Nitrogen-Doped Carbon Nanotube Confined Co–N_x_ Sites for Selective Hydrogenation of Biomass-Derived Compounds. Adv. Mater..

[32] Su Y., Chen C., Zhu X., Zhang Y., Gong W., Zhang H., Zhao H., Wang G. (2017). Carbon-embedded Ni nanocatalysts derived from MOFs by a sacrificial template method for efficient hydrogenation of furfural to tetrahydrofurfuryl alcohol. Dalton Trans..

[33] Ewald S., Standl S., Hinrichsen O. (2018). Characterization of nickel catalysts with transient methods. Appl. Catal. A.

[34] Nagakura S. (1957). Study of Metallic Carbides by Electron Diffraction Part I. Formation and Decomposition of Nickel Carbide. J. Phys. Soc. Jpn..

[35] Shi J., Hashiba Y., Nittono O. (2001). Preparation and characterization of Ni-C composite films. J. Mater. Sci..

[36] Kim S.-W., Son Y., Choi K., Kim S.-I., Son Y., Park J., Lee J.H., Jang J.-H. (2018). Highly Active Bifunctional Electrocatalysts for Oxygen Evolution and Reduction in Zn–Air Batteries. ChemSusChem.

[37] Xing J., Guo K., Zou Z., Cai M., Du J., Xu C. (2018). In situ growth of well-ordered NiFe-MOF-74 on Ni foam by Fe^2+^ induction as an efficient and stable electrocatalyst for water oxidation. Chem. Commun..

[38] Kovács G.J., Bertóti I., Radnóczi G. (2008). X-ray photoelectron spectroscopic study of magnetron sputtered carbon–nickel composite films. Thin Solid Films.

[39] Furlan A., Lu J., Hultman L., Jansson U., Magnuson M. (2014). Crystallization characteristics and chemical bonding properties of nickel carbide thin film nanocomposites. J. Phys. Condens. Matter..

[40] André R.F., Meyniel L., Carenco S. (2022). Nickel carbide (Ni3C) nanoparticles for catalytic hydrogenation of model compounds in solvent. Catal. Sci. Technol..

[41] Cao Y., Zhang H., Liu K., Zhang Q., Chen K.-J. (2019). Biowaste-Derived Bimetallic Ru–MoOx Catalyst for the Direct Hydrogenation of Furfural to Tetrahydrofurfuryl Alcohol. ACS Sustain. Chem. Eng..

[42] Jia P., Lan X., Li X., Wang T. (2019). Highly Selective Hydrogenation of Furfural to Cyclopentanone over a NiFe Bimetallic Catalyst in a Methanol/Water Solution with a Solvent Effect. ACS Sustain. Chem. Eng..

[43] Sunyol C., English Owen R., González M.D., Salagre P., Cesteros Y. (2021). Catalytic hydrogenation of furfural to tetrahydrofurfuryl alcohol using competitive nickel catalysts supported on mesoporous clays. Appl. Catal. B..

[44] Parikh J., Srivastava S., Jadeja G.C. (2019). Selective Hydrogenation of Furfural to Tetrahydrofurfuryl Alcohol Using Supported Nickel–Cobalt Catalysts. Ind. Eng. Chem. Res..

