# Supplementary material for: Reticular Coordination Induced Interfacial Interstitial Carbon Atoms on Ni Nanocatalysts for Highly Selective Hydrogenation of Bio-Based Furfural under Facile Conditions
Source: Nanomaterials (Basel). 2023 Jan 10;13(2):285. doi: 10.3390/nano13020285 (PMC9861954; doi:10.3390/nano13020285)
Supplement: Supplementary file 1 [file nanomaterials-13-00285-s001.zip › nanomaterials-2122342-supplementary.pdf]

nanomaterials

Supporting Information

Reticular Coordination Induced Interfacial Interstitial Carbon Atoms on Ni Nanocatalysts for Highly Selective Hydrogenation of Bio-Based Furfural under Facile Conditions

Dandan Liu *, Qiuju Fu, Chao Feng, Taisan Xiang, Han Ye, Yuting Shi, Liangjun Li, Pengcheng Dai, Xin Gu and Xuebo Zhao *

State Key Laboratory of Heavy Oil Processing, College of New Energy, College of Chemistry and Chemical Engineering, China University of Petroleum (East China), Qingdao, 266580, China; fqj_fuqiuju@163.com (Q.F.); fch_upc@163.com (C.F.); xiangtaisan@163.com (T.X.); yehan18zjou@163.com (H.Y.); sytgogogo1@163.com (Y.S.); lilj@upc.edu.cn (L.L.); dpcapple@upc.edu.cn (P.D.); guxin@upc.edu.cn (X.G.)
* Correspondence: liudandan@upc.edu.cn (D.L.); zhaoxuebo@upc.edu.cn (X.Z.)

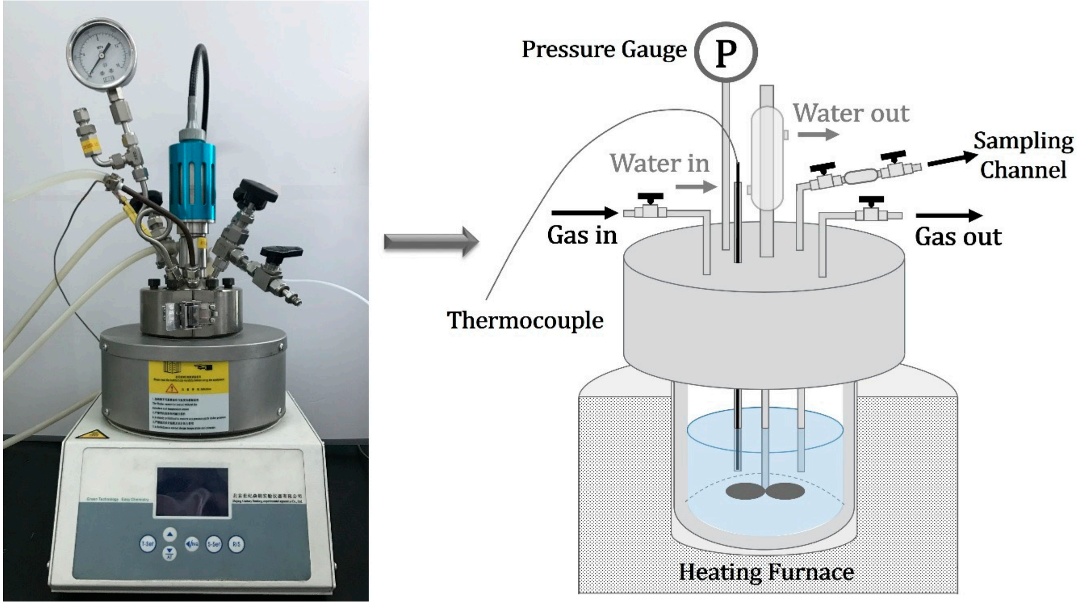

Figure S1. The apparatus of stainless steel batch reactor for catalyst performance evaluation of furfural hydrogenation.

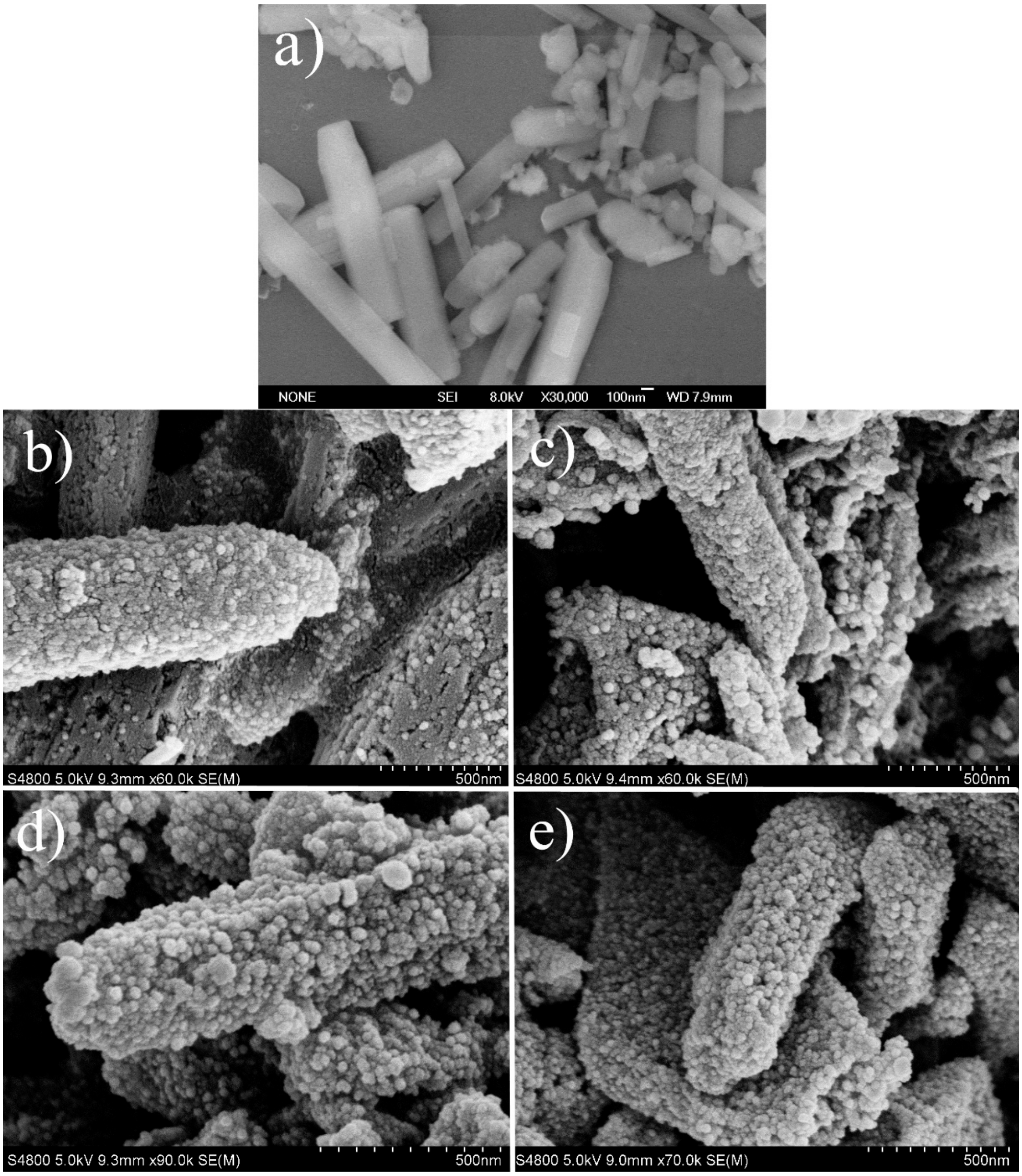

Figure S2. SEM images of MOF-74-Ni and derived Ni/C catalysts: **a)** MOF-74-Ni, **b)** Ni/C-300, **c)** Ni/C-400, **d)** Ni/C-500, **e)** Ni/C-600.

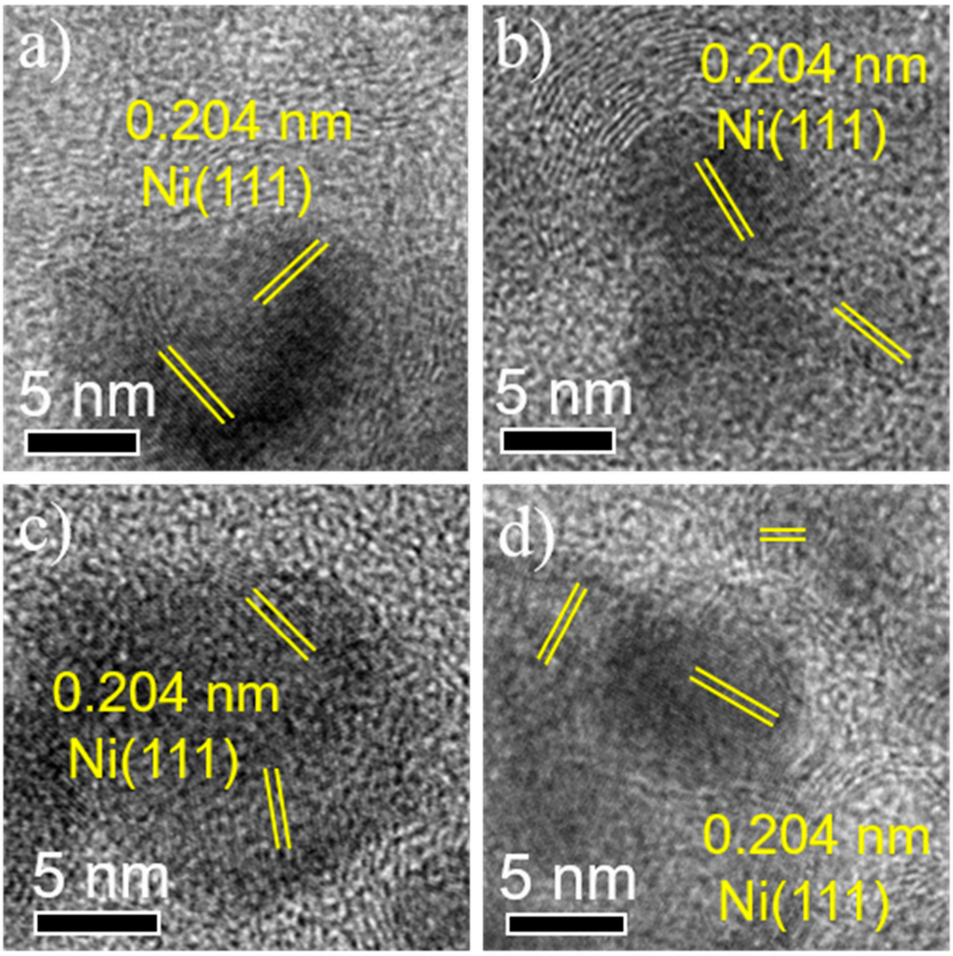

Figure S3. HRTEM images of Ni/C catalysts derived from MOF-74-Ni at different temperatures: **a)** Ni/C-300, **b)** Ni/C-400, **c)** Ni/C-500, **d)** Ni/C-600.

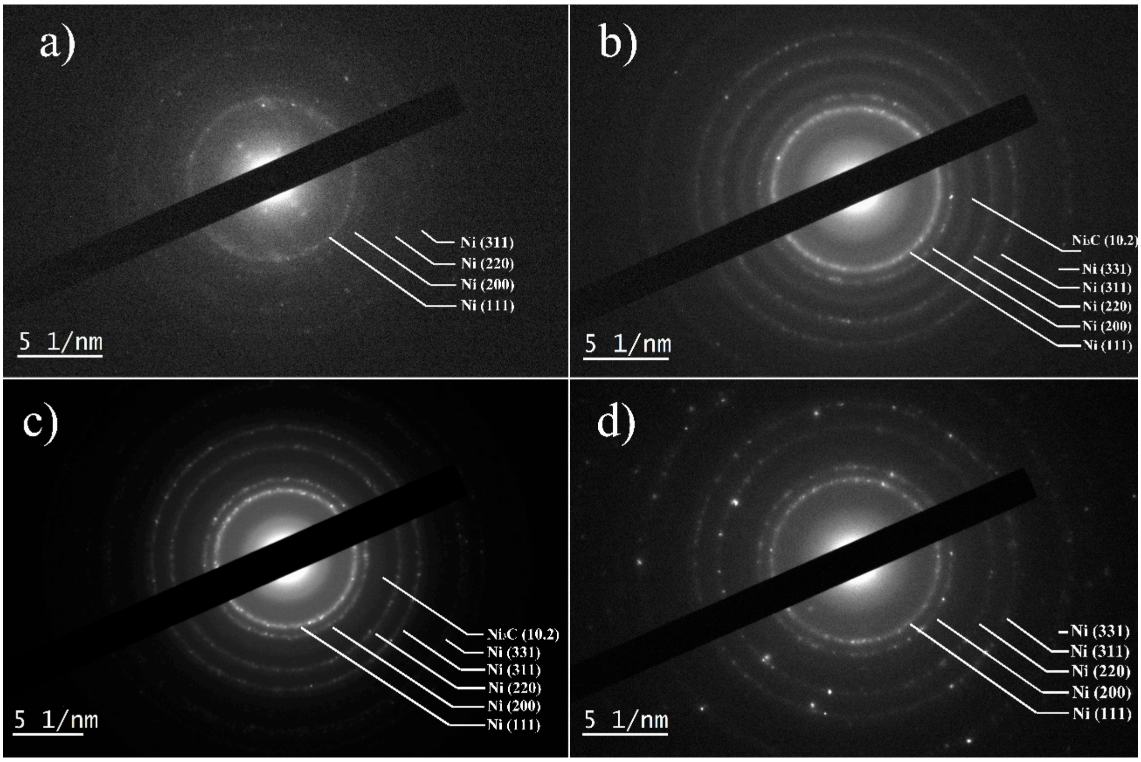

Figure S4. Electron diffraction patterns of Ni/C catalysts derived from MOF-74-Ni at different temperatures: **a)** Ni/C-300, **b)** Ni/C-400, **c)** Ni/C-500, **d)** Ni/C-600.

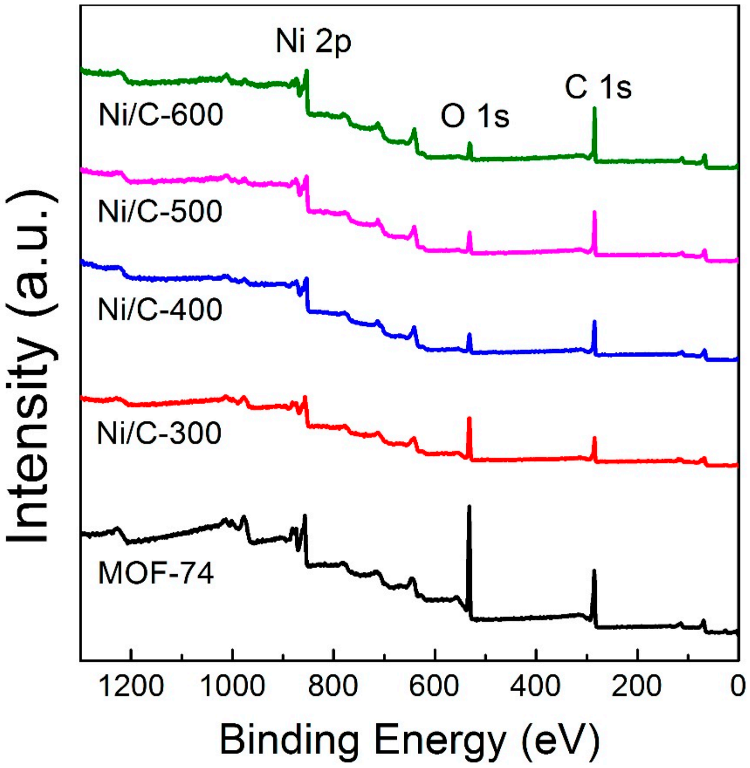

Figure S5. XPS survey spectra of the MOF-74 and Ni/C derivative prepared at different temperatures.

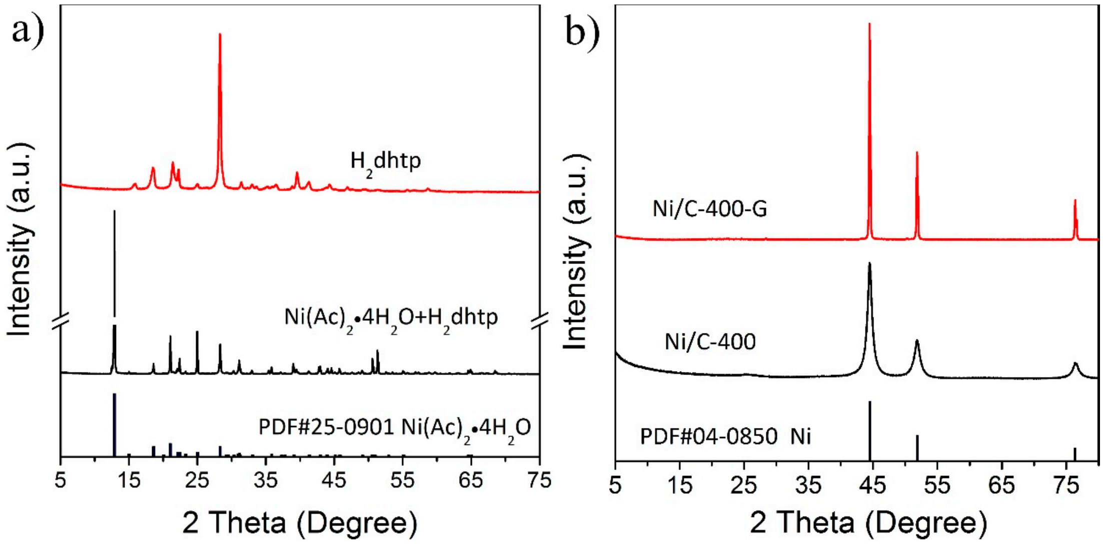

Figure S6. a) XRD patterns of mixture of $Ni(Ac)\cdot 4H_2O$ and H_2dhtp by physical grinding, and **b)** comparison of XRD patterns between Ni/C-400 and Ni/C-400-G.

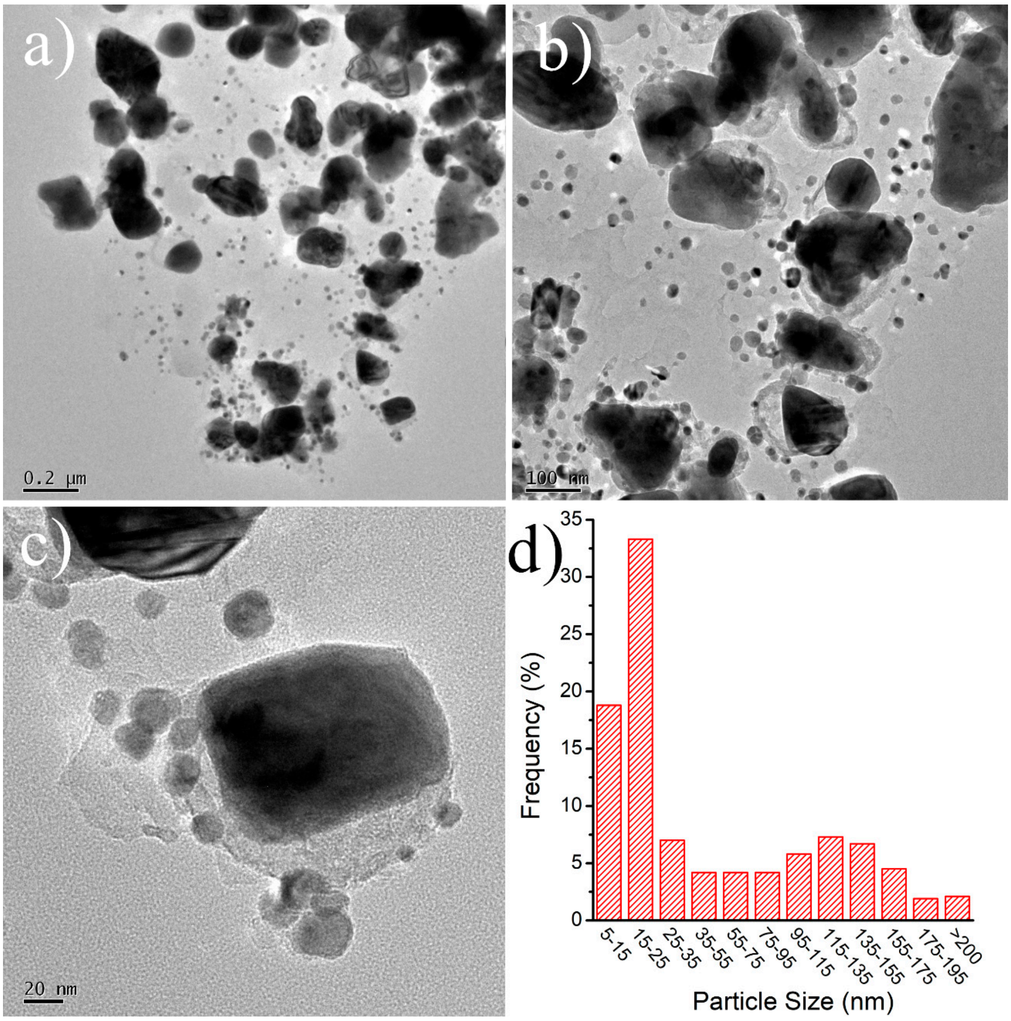

Figure S7. a)–c) TEM images of Ni/C-400-G catalyst with different magnifications, and **d)** corresponding particle size distribution.

Table S1. Characteristics of Ni-MOF-74 and Ni/C catalysts with different treatment temperatures.

Samples	Surface area (m^2 g^{-1}) S_{BET}	Surface area (m^2 g^{-1}) S_{Langm}	S_{micro} (m^2 g^{-1})	S_{meso} (m^2 g^{-1})	V_{meso} (cm^3 g^{-1})	V_{total} (cm^3 g^{-1})	D_{Pore} (nm)
MOF-74	1249	1337	1162	87	0.11	0.55	1.75
Ni/C-300	749	771	655	94	0.38	0.62	3.30
Ni/C-400	336	--	33	303	0.78	0.78	9.32
Ni/C-500	158	--	0	158	0.42	0.42	10.6
Ni/C-600	101	--	0	101	0.23	0.23	9.07

Table S2. XPS Data for the MOF-74 and Ni/C derivative prepared at different temperatures.

Sample	Atomic concentration /%			Ni^0		Ni-C		Ni^{2+}	
	C 1s	O 1s	Ni 2p	B.E.	At%	B.E.	At%	B.E.	At%
MOF-74	56.54	34.65	8.8	--	--	--	--	856.5	100
Ni/C-300	52.33	36.33	11.34	852.3	17.6	853.7	10.6	855.9	71.9
Ni/C-400	70.57	17.16	12.27	852.6	40.6	854.2	16.7	856.1	42.7
Ni/C-500	70.79	17.76	11.95	852.6	43.3	854.0	13.4	855.8	43.3
Ni/C-600	73.00	14.25	12.75	852.5	42.1	853.8	15.5	855.7	42.4

Table S3. C1s XPS Data for the MOF-74 and Ni/C derivative prepared at different temperatures.

Sample	C-C sp2		C-C sp3		C-O		O-C=O		Ni-C	
	B.E.	At %	B.E.	At %	B.E.	At %	B.E.	At %	B.E.	At %
MOF-74	284.6	51.7	--	--	286.2	21.8	288.5	26.5	--	--
Ni/C-300	284.6	53.0	285.6	7.7	286.2	17.9	288.9	21.4	--	--
Ni/C-400	284.6	59.4	285.6	14.5	286.6	10.2	288.9	13.1	283.8	2.8
Ni/C-500	284.6	60.1	285.7	8.0	286.5	10.8	289.1	15.8	283.8	5.3
Ni/C-600	284.6	60.4	285.5	6.5	286.5	10.8	289.1	15.7	283.9	6.6

Table S4. The physicochemical properties of Ni/C nanocatalysts obtained at different temperatures.

Catalyst	Ni^a (wt %)	d_{TEM}^b (nm)	S_{Ni}^c (m^2/g)	D^d (%)
Ni/C-300	35.5	8.21	4.61	1.95
Ni/C-400	39.1	8.58	18.61	7.17
Ni/C-500	38.2	11.9	3.79	1.50
Ni/C-600	40.4	14.7	3.14	1.15

a Determined by XPS. b Particle size determined from TEM. c Metal surface area calculated on the basis of the amount of H_2 consumption in H_2 impulse. d. Dispersion calculated from specific area of Ni.

Table S5. The Bader charge analysis of a) Ni and b) Ni with interstitial carbon.

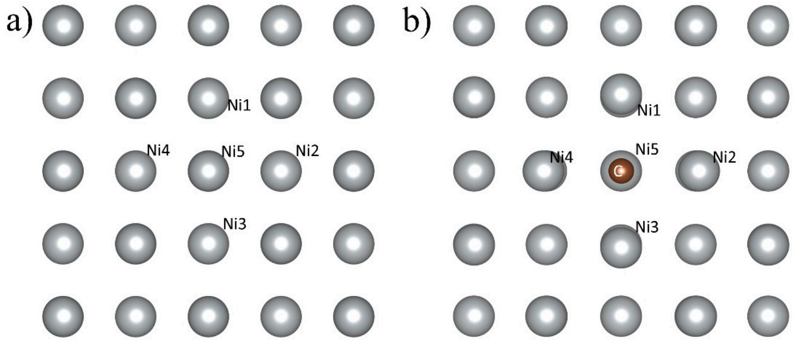

Atom	Ni1	Ni2	Ni3	Ni4	Ni5	C
a) Ni	9.994999	9.998755	10.00627	9.998755	9.998755	--
b) Ni-C	9.914927	9.853855	9.858288	9.853855	9.8537	4.822521

Table S6. The catalytic performance of Ni/C-400 and Ni/C-400-G.

Catalyst	Temperature (°C)	H_2 Pressure (MPa)	Time (h)	Conversion (%)	Selectivity (%)		
					FA	THFA	Others
Ni/C-400	80	3	4	> 99	0.1	97.7	2.2
Ni/C-400-G	80	3	4	3.3	5	4.9	90.1

Reaction conditions: 0.6 g FFR, 0.1 g catalysts, 50 mL ethanol; Others: refer to byproducts mainly contain 2-furaldehyde diethyl acetal and other not detected.

Table S7. Summary for hydrogenation of furfural to tetrahydrofurfuryl alcohol from literatures.

Feedstocl	Catalyst	Solvent	m_{cat}/m_{FFR}	Conditions	Conv. /%	S_{THFA}/%	Refs
Furfural	Pd/C	Isopropano l	1:100	180 °C, 2 MPa, 5 h	100	64	[1]
Furfural	Pd/Al_2O_3	Isopropano l	1:20	25 °C, 6 MPa, 8 h	79.5	100	[2]
Furfural	Ru–MoO_x/C N	Water	1:2.42	100 °C, 2 MPa, 1 h	91.7	99	[3]
Furfural	Pd–Ni/MWNT	Ethanol	1:5.8	100 °C, 3 MPa, 5 h	92.7	78.2	[4]
Furfural	Pt(3)Ni(3)/C	Water	1:2	35 °C, 2 MPa, 12 h	99	93	[5]
Furfural	NiFe/SBA-15	Methanol: water = 3:1	1:4	140 °C, 3.4 MPa, 5 h	99.8	95.8	[6]
Furfural	Ni-Co/SBA-15	Isopropano l	1:6.6	210 °C, 7 MPa, 6 h	100	90.4	[7]
Furfural	Cu_1Ni_3/MgAlO	Ethanol	1:9.6	150 °C, 4 MPa, 3 h	99	95	[8]
Furfural	Ni@NCNTs	Water	1:3.2	100 °C, 4 MPa, 7 h	100	99.5	[9]
Furfural	Ni/C(Ni-BTC	Isopropano l	1:1	120 °C, 1 MPa, 2 h	100	100	[10]
Furfural	Ni/MMO-CO	Isopropano l	1:5.8	110 °C, 3 MPa, 3 h	100	99	[11]
Furfural	Ni(40)/MgO(3 0)-M	Water	1:2.5	140 °C, 4 MPa, 4 h	100	99	[12]
Furfural	Ni/C(Ni-BTC	Ethanol	1:67.2	150 °C, 3 MPa, 18 h	86.7	81.3	[13]
Furfural	$NiCu_{0.33}$/C	Ethanol	1:67.2	150 °C, 3 MPa, 18 h	99.9	94.6	[13]
Furfural	Ni/C-400	Ethanol	1:6	80 °C, 3 MPa, 4 h	> 99	> 96	This work

Reference

1. Nguyen-Huy, C.; Kim, J.S.; Yoon, S.; Yang, E.; Kwak, J.H.; Lee, M.S.; An, K. Supported Pd nanoparticle catalysts with high activities and selectivities in liquid-phase furfural hydrogenation. *Fuel* **2018**, *226*, 607–617.
2. Bhogeswararao, S.; Srinivas, D. Catalytic conversion of furfural to industrial chemicals over supported Pt and Pd catalysts. *J. Catal.* **2015**, *327*, 65–77.
3. Cao, Y.; Zhang, H.; Liu, K.; Zhang, Q.; Chen, K.-J. Biowaste-Derived Bimetallic Ru–MoOx Catalyst for the Direct Hydrogenation of Furfural to Tetrahydrofurfuryl Alcohol. *ACS Sustain. Chem. Eng.* **2019**, *7*, 12858–12866.
4. Liu, L.; Lou, H.; Chen, M. Selective hydrogenation of furfural over Pt based and Pd based bimetallic catalysts supported on modified multiwalled carbon nanotubes (MWNT). *Appl. Catal. A.* **2018**, *550*, 1–10.
5. Wu, J.; Zhang, X.; Chen, Q.; Chen, L.; Liu, Q.; Wang, C.; Ma, L. One-Pot Hydrogenation of Furfural into Tetrahydrofurfuryl Alcohol under Ambient Conditions over PtNi Alloy Catalyst. *Energ. Fuels* **2020**, *34*, 2178–2184.
6. Jia, P.; Lan, X. Li, X.; Wang, T. Highly Selective Hydrogenation of Furfural to Cyclopentanone over a NiFe Bimetallic Catalyst in a Methanol/Water Solution with a Solvent Effect. *ACS Sustain. Chem. Eng.* **2019**, *7*, 15221–15229.
7. Parikh, J.; Srivastava, S.; Jadeja, G.C. Selective Hydrogenation of Furfural to Tetrahydrofurfuryl Alcohol Using Supported Nickel–Cobalt Catalysts. *Ind. Eng. Chem. Res.* **2019**, *58*, 16138–16152.
8. Wu, J.; Gao, G.; Li, J.; Sun, P.; Long, X.; Li, F. Efficient and versatile CuNi alloy nanocatalysts for the highly selective hydrogenation of furfural. *Appl. Catal B* **2017**, *203*, 227–236.
9. Gong, W.; Chen, C.; Zhang, H.; Wang, G.; Zhao, H. Highly dispersed Co and Ni nanoparticles encapsulated in N-doped carbon nanotubes as efficient catalysts for the reduction of unsaturated oxygen compounds in aqueous phase. *Catal. Sci. Technol.* **2018**, *8*, 5506–5514.
10. Su, Y.; Chen, C.; Zhu, X.; Zhang, Y.; Gong, W.; Zhang, H.; Zhao, H.; Wang, G. Carbon-embedded Ni nanocatalysts derived from MOFs by a sacrificial template method for efficient hydrogenation of furfural to tetrahydrofurfuryl alcohol. *Dalton Trans.* **2017**, *46*, 6358–6365.
11. Meng, X.; Yang, Y.; Chen, L.; Xu, M.; Zhang, X.; Wei, M. A Control over Hydrogenation Selectivity of Furfural via Tuning Exposed Facet of Ni Catalysts. *ACS Catal.* **2019**, *9*, 4226–4235.

12. Sunyol, C.; English Owen, R.; González, M.D.; Salagre, P.; Cesteros, Y. Catalytic hydrogenation of furfural to tetrahydrofurfuryl alcohol using competitive nickel catalysts supported on mesoporous clays. *Appl. Catal. B.* **2021**, *611*, 117903
13. Tang, F.; Wang, L.; Dessie Walle, M.; Mustapha, A.; Liu, Y.-N. An alloy chemistry strategy to tailoring the d-band center of Ni by Cu for efficient and selective catalytic hydrogenation of furfural. *J. Catal.* **2020**, *383*, 172–180.
